# Multidisciplinary team (MDT) meeting and Radiologist workload, a prospective review in a tertiary care hospital

**DOI:** 10.12669/pjms.336.12905

**Published:** 2017

**Authors:** Sadaf Nasir, Saleha Anwar, Moinuddin Ahmed

**Affiliations:** 1 Sadaf Nasir, MBBS, FCPS.Department of Radiology, Liaquat National Hospital, National Stadium Road, Karachi, Pakistan; 2 Saleha Anwar, MCPS, FCPS.Department of Radiology, Liaquat National Hospital, National Stadium Road, Karachi, Pakistan; 3 Moinuddin Ahmed, FCPS. Department of Radiology, Liaquat National Hospital, National Stadium Road, Karachi, Pakistan

**Keywords:** Multidisciplinary team meeting (MDTM), Radiology Workload

## Abstract

**Objective:**

To quantify the increase in workload associated with multidisciplinary team meetings for radiologists in a tertiary care hospital over a period of 15 months.

**Methods:**

Data was collected prospectively regarding number of multidisciplinary team meetings, number of clinical cases discussed, number of individual imaging studies reviewed, and preparation time of residents, senior registrar and consultants and the delivery time of meeting.

**Results:**

Total 223 meetings were held over 15 months (April 2014 to June 2015) for 12 clinical specialty areas. There were 1120 clinical case discussions and a total of 2759 documented individual imaging studies reviewed. Resident’s preparation time was 74.6 hours/month, senior registrar’s preparation time was 47.93 hours/month, consultant’s preparation time was 18.67 hours/month and the total duration time for meetings was 18 hours/month.

**Conclusion:**

Multidisciplinary team meetings now represent a significant workload of radiology and has reduced the time for other academic activities within the department.

## INTRODUCTION

Multidisciplinary team meetings (MDTMs) are a structured medium of approach to healthcare where a multifaceted team of professionals work concertedly to strategize a treatment plan for a patient.^1^-^4^ This team comprises of all the subspecialisties required to take a case from diagnosis to treatment to functional rehabilitation of the patient and thereby includes medical as well as allied health professionals such as physiotherapists and occupational therapists etc.^3^,^5^-^7^ Such a modus operandi is applied in an effort to put together the best minds and bring forth the most effective clinical arsenal for the benefit of the patient.^2^,^8^,^9^ Throughout the years MDTMs have been most beneficial in meticulous diagnosis and treatment leading to better outcomes and ultimately greater patient satisfaction.^1^,^2^,^9^,^11^ Furthermore, these meetings prove to be valuable platforms for learning and discussing challenging cases.^11^

However, MDTMs themselves necessitate a great deal of organization, management infrastructure and funding to ensure the presence of relevant personnel, the collection and compilation of important patient details and radiological materials.^2^,^8^,^11^,^12^ The Radiology department plays a pivotal and unequivocal role in all such meetings since it is the quintessential visual discipline of modern medicine.^2^,^13^ The Radiologists must invest their time and energy as well as resources while preserving a professional decorum; so it is only plausible that the amplified workload and increasing pressures so often takes its toll.^2^,^14^ The aim of this study was to highlight the increase in workload and time consumption associated with MDTMs for radiologists working in a tertiary care center.

## METHODS

The study was conducted prospectively at Liaquat National Hospital, Department of Radiology, for duration of 15 months; from April 2014 to June 2015.

MDTMs at Liaquat National Hospital are scheduled monthly. The list of cases to be discussed is circulated between the participants prior to the meetings. The case files, particularly radiological investigations, are initially reviewed by the residents, who study the material and gather the relevant information of the patients (e.g. past history, clinical diagnosis etc.). The data is then compiled and submitted to the registrars and the consultants for further discussion and proofreading. All the radiological images are reviewed on PACS and in case of imaging performed elsewhere, the films are reviewed.

For the purpose of this study, the senior radiology faculty and residents were asked to note the time they spent in preparation of the meetings and keep a monthly record as accurate as possible. Data was collected regarding the number of MDTMs scheduled and held, total number of cases and individual imaging studies, number of images quickly reviewed (within 1-5 mins/image), number of imaging studies not discussed, preparation time (hours/month) of residents, senior registrar and consultants, and the total duration time(hours/month) of the meetings.

## RESULTS

An overview of meeting schedule, preparation material and the mean number of patients discussed is shown in Table-I. There were approximately 14 meetings scheduled per month from the period of April 2014 to December 2014. Additional three meetings started per month from January 2015 with a total of 17 meetings per month. A total of 228 meetings were scheduled over a period of 15 months and 223 held. five MDTs were cancelled due to key personnel unavailability. There were a total of 1120 clinical case discussions (mean=74.66/month) and a total of 2759 documented individual imaging studies were reviewed (mean183.93/month). Those situations were reported where images might be quickly reviewed, might not be considered relevant to the discussion or might not be presented due to time constraints and hence would not be presented in the meeting. At least one consultant radiologist and one registrar were required to be present at each meeting. The residents, in contrast attended all the meetings.

**Table-I:** Data of monthly meetings and cases/images discussed.

*Month*	*Number of meetings scheduled*	*Number of meetings held*	*Total number of cases/ patient’s*	*Total number of individual imaging studies*	*Number of images quickly reviewed*	*No of imaging studies not discussed*
April 2014	14	14	65	107	Nil	03
May 2014	14	13	63	99	Nil	Nil
June 2014	14	14	77	118	03	04
July 2014	14	14	68	112	02	02
Aug 2014	14	12	54	95	Nil	Nil
Sep 2014	14	14	71	103	Nil	Nil
Oct 2014	14	14	65	128	Nil	05
Nov 2014	14	14	70	132	Nil	Nil
Dec 2014	14	14	72	121	05 `	06
Jan 2015	17	16	84	134	03	04
Feb 2015	17	17	91	147	06	Nil
Mar 2015	17	17	79	136	Nil	03
April 2015	17	16	82	145	Nil	05
May 2015	17	17	89	151	Nil	Nil
June 2015	17	17	90	137	02	03

The time spent in preparation of and at the meetings for each month is summarized in Table-II. Residents preparation time was 1119 hours (mean=74.6 hours/month), senior registrar’s preparation time was 719 hours (mean=47.93 hours/month) and consultants preparation time was 280 hours mean=18.67 hours/month). Duration of meetings was 270 hours (mean= 18 hours/month).

**Table-II:** Individual contribution on behalf of Radiology team.

*Month*	*Resident’s preparation time (hours)*	*Senior registrar’s preparation time (hours)*	*Consultant’s preparation time (hours)*	*Total delivery time of the meetings (hours)*
April 2014	64.2	42.8	17.2	17.5
May 2014	59.4	39.6	14.8	16.25
June 2014	70.8	47.2	17.7	16.1
July 2014	67.2	44.8	16.8	16.8
Aug 2014	57	33.2	14.3	15
Sep 2014	61.8	38.1	15.4	15.7
Oct 2014	76.8	51.2	19.2	17
Nov 2014	79.2	47.5	18.8	18.2
Dec 2014	72.6	44.7	18.1	17.08
Jan 2015	80.4	49.6	20.1	20
Feb 2015	88.2	58.8	22.0	21.25
Mar 2015	81.6	50.3	19.4	19.55
April 2015	87	56	21.7	18.4
May 2015	90.6	60.4	22.6	21.25
June 2015	82.2	54.8	21.9	20

Statistical analysis was done by using SPSS (Statistical Package of Social Sciences) version 17.0. Descriptive statistics were calculated. For quantitative variable Pearson correlation and t–test (as applicable) were applied. The p-value ≤ 0.05 was considered as significant.

Among total study participant, 53.3% had ≤74 cases while 46.7% had >74 cases. As far as images for study are concerned, 46.7% have ≤124 images for study and 53.3% had >124 images for study (Table-III). The overall mean number of study cases was 74.66±11.04. The mean number of images for study was 124.33±18.07. The mean number of scheduled meetings was 15.20±1.52 and mean number of meetings held was 14.86±1.64. The overall mean resident preparation time (hours) was 74.60±10.84. This time for senior registrar was 47.93±7.72 hours. But the mean preparation time for consultants was 18.66±2.68 hours. The mean total duration time of cases during meetings was 18.00±2.00hours (Table-IV).

**Table-III:** Frequency Distribution for Number of Cases and Images for study.

	*n*	*%*
***Total number of cases***		
≤74	8	53.3
>74	7	46.7
***Total number of Imaging Studies***		
≤124	7	46.7
>124	8	53.3

**Table-IV:** Descriptive statistics of number of cases, images for study, preparation time and meeting time.

	*Mean ± S.D*
Number of Cases	74.66±11.04
Number of images for study	124.33±18.07
Number of Meetings Scheduled	15.20±1.52
Number of Meetings Held	14.86±1.64
Resident Preparation Time Hours	74.60±10.84
Senior Registrars Preparation Time Hours	47.93±7.72
Consultant Preparation Time Hours	18.66±2.68
Total Duration Time of Meetings Hour	18.00±2.00

The correlation results were presented in Table-V. The results showed moderately significant correlation of number of cases with resident preparation time (r=0.747), senior registrar’s preparation time (r=0.744), consultant preparation time hours (r=0.76) and total duration of meetings r=0.726). Number of images for study also showed moderately significant correlation with resident preparation time (r=0.882), senior registrar’s preparation time (r=0.844), consultant preparation time (r=0.801) and total duration of meetings (r=0.726).

**Table-V:** Correlation of number of cases and number of images for study with preparation time and meeting time.

	*Total number of cases*	*Total numer of individual imaging studies*

	*r-values*	*r-values*
Resident Preparation	0.747	0.882
Time Hours	p=0.001*	p<0.001*
Senior Registrars	0.744	0.808
Preparation	p=0.001*	p<0.001*
Time Hours		
Consultant Preparation	0.76	0.844
Time Hours	p=0.001*	p<0.001*
Total Duration Time of Meetings Hour	0.726 p=0.002*	0.801 p<0.001*

Pearson correlation is applied,

* Significant at ≤ 0.05

The comparison of mean of resident preparation time, senior registrar’s preparation time, consultant preparation time and total duration of meetings was done with respect to stratified groups of number of cases (≤74 cases and >74 cases) and number of images for study (≤124 cases and >124 cases). The results are also presented in Table-VI and Table-VII. A statistically significant difference was observed with resident preparation time (p=0.001), senior registrar’s preparation time (p=0.001), consultant preparation time (p=0.001), and total duration of meetings hours (p=0.002) between the stratified groups of number of cases. A statistically significant difference was observed between the stratified groups of number of images for study with resident preparation time (p<0.001), senior registrar’s preparation time (p<0.001), consultant preparation time hours (p<0.001) and total duration of meetings (p=0.002).

**Table-VI:** Comparison of mean preparation and meeting time between number of cases in various groups

	*Number of Cases*		*p-value*

	*≤74*	*>74*	
Resident Preparation Time Hours	67.27±8.17	82.97±6.57	0.001*
Senior Registrars Preparation Time Hours	42.73±5.66	53.87±4.96	0.001*
Consultant Preparation Time Hours	16.82±1.84	20.77±1.76	0.001*
Total Duration Time of Meetings Hour	16.69±1.01	19.50±1.79	0.002*
Independent T-test is applied			

* Statistically Significant at ≤ 0.05

**Table-VII:** Comparison of mean preparation and meeting time between number of images for study.

	*Total No of Individual imaging studies*		*p-value*

	*≤124*	*>124*	
Resident Preparation Time Hours	64.71±5.80	83.25±4.81	<0.0001*
Senior Registrars Preparation Time Hours	41.48±4.81	53.57±4.63	<0.0001*
Consultant Preparation Time Hours	16.32±1.48	20.71±1.49	<0.0001*
Total Duration Time of Meetings Hour	16.34±0.85	19.45±1.50	<0.0001*
Independent T-test is applied			

* Statistically Significant at ≤ 0.05

## DISCUSSION

With the passage of time, due to advancement in health care facilities the multidisciplinary meetings have emerged playing a pivotal role in important decision making in the treatment of patient.^2^

Over the years it has been noticed that these meetings have multiplied. There is a continuous demand for these meetings to be increased. The need for review of pathology and radiology findings during discussion speaks for the success and importance of these meetings.^2^,^6^,^10^ These meetings also serve as a platform for learning. Due to extensive advancement in the field of radiology over the past years, the decisions for making the choice of radiological procedure has become complex now. These meetings are playing a very important role in professional development of the radiologists and also are a major contributor in the decision making for critically ill patients.^15^,^16^

However due to increase in workload in the department of radiology in terms of increase in number of patients, it is difficult for the faculty to cope up with all the clinicopathological meetings. There are some major issues with fulfilling the demand of these meetings. It includes the time spent in preparation of these meetings, the number of residents and senior registrar engaged during the preparation besides the time spent during the meeting.

The increase in number of meetings has placed extra pressure on the radiology department. The residents’ pre meeting preparation time has increased from 64.2 hour/month (April 2014) to 82.2 hour/month (June 2015). The consultant preparation time has also increased from 17.2hours / month to 21.9 hours / month. This increase workload has resulted in increased working hours by both faculty and residents.

Radiology is a service providing department with patient inflow from both outpatient and inpatient departments. Most of the time it`s difficult to accommodate the preparation of these meetings in the usual working hours of the department. This usually impacts the workflow of the department, which is very high paced. Most of these preparations are either done during late working hours or in early mornings. Up till now this is being done due to professional attitude and by having a strong sense of commitment with the patient. There is no compensation model approved for members of the MDTMs.^2^

Another issue, which is frequently faced by radiologist, is the review of outside source images. These are of different quality and not necessarily according to standardized protocols. Much time is needed to review these films and then to make an assessment. Due to introduction of PACS the lives of radiologists have become a lot easier. It facilitates in the reviewing of images. It also helps in reducing the time spent on data retrieval and hence time spent in preparation of these meetings.

It is also noted that each multidisciplinary team wishes to conduct these meetings according to their availability. This becomes a problem for the radiology department, as they have to provide service to the patients and also run their own residency program. It is often troublesome to accommodate these meetings in the already scheduled academic Rota. Most of the times it`s not possible to suit everyone’s schedule that is involved in these meetings.

Another issue noted during these meetings is lack of coordination between the radiologists and the physicians. It was noted that more time was spent on cases in which there was incomplete clinical information provided to the radiologist.

## CONCLUSION

The multidisciplinary meetings add quality to the patient’s care and management. It has now become the integral part of patient’s management. The work and timings involved in the preparation and conduction of these meetings is increasing day by day. This has taken a lot of time of radiologist, who has to keep the departmental workflow in progress as well. It is predicted that these meetings would substantially increase in the future as most of the decision-making is being done in these valuable meetings. In order to keep the efficiency of these meetings the role of radiologist is pivotal. Efforts should be made to reduce the effects of workload and stress over the radiologist by taking measures such as incorporating these meetings during the work hours, by increasing the strength of department and by proper coordination between physicians and radiologists.

### Authors’ contribution

**SN** main conception and design with acquisition and analysis of manuscript.

**SN, SA and MUA** did data collection and manuscript writing.

**SA, MUA** preparation of the manscript, review and final approval of manuscript.
